# Quality indicators for the rehabilitation before and after total knee arthroplasty in Japan: a modified Delphi method and practice test

**DOI:** 10.1186/s42836-024-00297-5

**Published:** 2025-03-06

**Authors:** Yoshinori Hiyama, Masashi Taniguchi, Shosuke Ohtera, Osamu Wada, So Tanaka, Masato Kako

**Affiliations:** 1 Department of Physical Therapy, Faculty of Health Science, Juntendo University, 2-1-1 Hongo, Bunkyo-Ku, Tokyo, 113-8421 Japan; 2 Graduate School of Medicine, Human Health Sciences, Kyoto University, 53-Kawahara-ChoSakyo-Ku, ShogoinKyoto, 606-8507 Japan; 3 Department of Health Economics, Center for Gerontology and Social Science, National Center for Geriatrics and Gerontology, Research Institute, 7-430 Morioka-Cho, Obu City, Aichi, 474-8511 Japan; 4 Anshin Hospital, 1-4-12 Minatojima-Minamimachi, Chuo-Ku, Kobe City, Hyogo, 650-0047 Japan; 5 Fukuoka Orthopaedic Hospital, 2-10-50 Yanagochi, Minami-Ku, Fukuoka City, Fukuoka, 815-0063 Japan; 6 Toyota Memorial Hospital, 1-1 Heiwacho, Toyota City, Aichi, 471-8513 Japan

**Keywords:** Knee osteoarthritis; Total knee arthroplasty; Rehabilitation; Quality indicator

## Abstract

**Background:**

It is important to adhere to the pertinent guidelines to ensure evidence-based rehabilitation of patients with total knee arthroplasty (TKA); however, studies have suggested that pre- and post-TKA rehabilitation provided in Japan may not be adequately evidence-based. Quality indicators (QIs) translate practice guidelines into actionable and measurable statements by identifying the clinical context, timing, and target population. This study aimed to develop QIs for pre- and post-TKA rehabilitation in Japan. Additionally, a pilot practice test was conducted to assess the feasibility and applicability of the developed QIs prior to their actual clinical application.

**Methods:**

This study used a modified Delphi technique (RAND/UCLA Appropriateness Method). A nine-member panel of clinicians and researchers evaluated the 49 proposed QIs related to rehabilitation before and after TKA. Panelists independently rated the 49 candidate QIs on a 9-point Likert scale and discussed these QIs in an online meeting. After the meeting, the panelists independently re-rated the QIs, and QIs with a median score of 7 or higher and score of less than 3 by two or fewer panelists were adopted as the final QIs. In addition, a pilot practice test was conducted to assess the feasibility and applicability of the developed QIs by retrospectively analyzing the medical records at two hospitals.

**Results:**

Forty-nine candidate QIs were developed based on one set of QIs, nine practice guidelines, eight best practice recommendations, and 162 systematic reviews. Finally, 36 indicators, including two new ones, were adopted consensually by nine panelists. Among these 36 indicators, some had overlapping elements, so they were consolidated and organized into 24 indicators. The pilot test (*n* = 352) revealed a median QI performance of 86.1 (IQR, 56.1–100), with six QIs demonstrating performance levels below 10%. This low performance indicated that the proportion of patients receiving rehabilitation in accordance with the indicators was actually low.

**Conclusions:**

This study developed 36 QIs for patients undergoing rehabilitation before and after TKA in Japan. Although their feasibility was confirmed at two facilities, future studies are warranted to measure the quality of care more comprehensively.

## Background

Total knee arthroplasty (TKA) is an effective treatment that improves knee pain and functional decline in patients with knee osteoarthritis [1]; 82,304 knee arthroplasties were performed in Japan in 2017 [2]. As recommended by several practice guidelines [3–5], pre- and postoperative rehabilitation plays a significant role in enhancing the benefits of TKA, including reducing pain before surgery [6], and improving knee function [7] and functional ambulation after surgery [8]. The proportion of patients with improvement in the activities of daily living was reportedly higher after the introduction of guideline-based care after TKA than before such care was introduced [9], highlighting the importance of guideline adherence and evidence-based rehabilitation.

In Japan, 29.2% of physical therapists use practice guidelines [10] against 54% of their counterparts in other countries who choose the treatment method recommended by the practice guidelines [11]. While 93% of the facilities in other countries provide the recommended rehabilitation for knee arthroplasty [11], more than 64.8% of facilities in Japan routinely use continuous passive motion (CPM) [12], which is not recommended [3], and only 45.4% of facilities offer the recommended preoperative rehabilitation, suggesting that rehabilitation before and after knee arthroplasty may not be adequately evidence-based in Japan.

A quality indicator (QI) defines the minimum standard of care and serves as a tool to measure and quantify healthcare processes, outcomes, patient perceptions, and organizational structure and/or systems related to the ability to provide high quality healthcare [13]. QIs translate practice guidelines into actionable and measurable statements by identifying the clinical context, timing, and target population [14]. This facilitates objective measurement and determination of whether the process meets the quality standards, with less room for interpretation than practice guidelines. Therefore, the development of QIs for rehabilitation before and after TKA could render the practice guidelines for pre- and post-TKA rehabilitation actionable and ensure evidence-based rehabilitation before and after TKA.

Four QI sets for TKA are available [15–18], including a rehabilitation-specific set [18], that proposes implementation of the following measures for minimum rehabilitation: pain [15, 18], gait ability [15, 18], range of motion of the knee joint [15, 18], muscle strength [15, 18], early mobilization [17, 18], and the knee outcome score [17, 18]. Although these indicators are useful for pre- and post-TKA rehabilitation, the average length of hospital stay after TKA in Japan is 21 days [19], which is substantially longer than the corresponding period of 4.4 days in Canada [20], where these indicators were developed. Therefore, the development of QIs is necessary because differences in the length of hospital stay may lead to differences in the role of rehabilitation during hospitalization. The QIs developed in this study, which consider the length of hospital stay, may be useful in countries with longer hospital stays than Canada, such as South Korea (13 days) [21] and China (9.3 days) [22].

Therefore, this study aimed to develop QIs for pre- and post-TKA rehabilitation in Japan and conduct a pilot practice test to assess the feasibility and applicability of the developed QIs prior to actual clinical measurement.

## Methods

The widely used multistep approach, modified Delphi technique (RAND Corporation [RAND]/University of California, Los Angeles [UCLA] Appropriateness Method) [23], was employed.

**Fig. 1:**
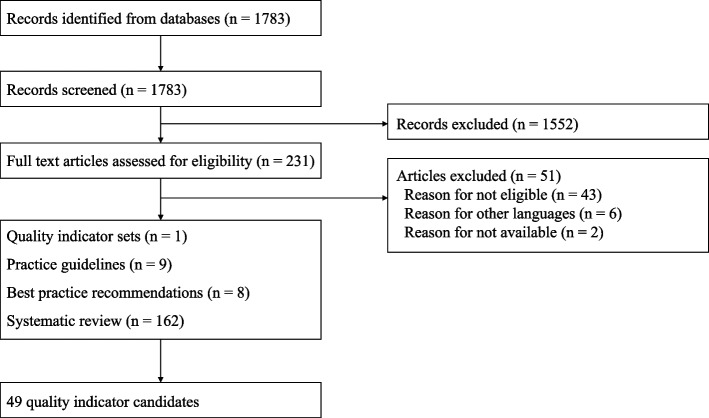
Flow chart of the literature search

Initially, a literature search was conducted for the available clinical practice guidelines, QIs, and systematic reviews published in English or Japanese on rehabilitation before and after TKA. An electronic database search was conduced by using the following keywords: “total knee arthroplasty” or “total knee replacement,” and “rehabilitation,” “exercise,” “physical therapy,” “postoperative care,” or “treatment” in September 2020. Two guideline databases (PEDro and National Institute for Health and Care Excellence [NICE] Find Guidance) and three databases (MEDLINE, CINAHL, and ICHUSHI) were searched. Two independent reviewers assessed the quality of the practice guidelines using the AGREE II instrument [24], the quality of QIs using the AGREE II-QI instrument [25], and the quality of systematic reviews using the AMSTAR tool [26] and reached consensus through discussion. Finally, one set of QIs, nine practice guidelines, eight best practice recommendations, and 162 systematic reviews (SRs) were used to develop the initial 49 QIs (Fig. 1).

### Expert panel

The panelists were included by taking into account age, experience, number of TKA surgeries performed, and length of hospital stay at their institutions to ensure diversity. They were from the following regions: Hokkaido, Tohoku, Kanto, Chubu, Kinki, Chugoku/Shikoku, and Kyusyu. Nine of the 10 recruited experts agreed to participate and provided informed consent. The panelists included an orthopedic surgeon, a researcher, and physical therapists.

### Building consensus

**Fig. 2:**
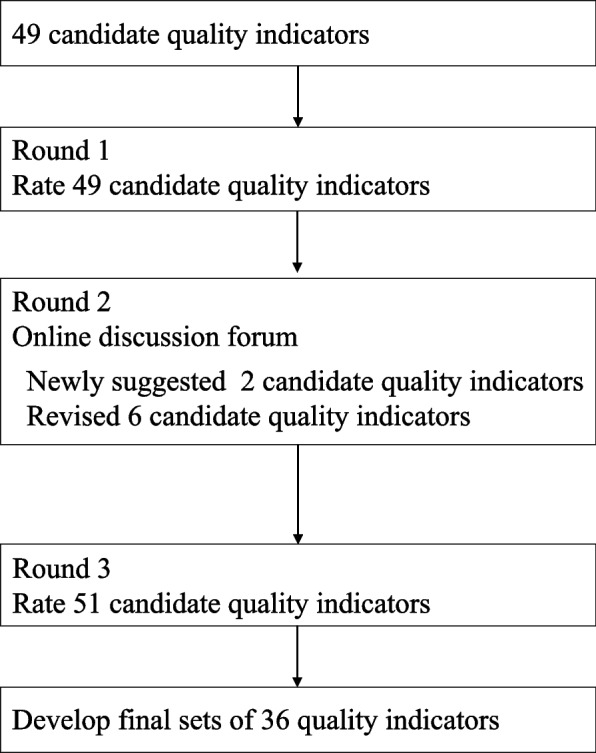
Flow chart of the modified Delphi process used to develop rehabilitation quality indicators

The modified Delphi process consisted of three rounds. In round 1, the panelists independently rated 49 candidate QIs and then discussed these QIs in the round 2, a meeting held online. In round 3, the panelists independently rated the candidate QIs again via discussion to determine the final QIs (Fig. 2).

In round 1, each panelist independently rated the 49 initial candidate QIs on a 9-point Likert scale, adapted from the following nine domains [27]: evidence-based, interpretable, actionable, denominator, numerator, validity, reliability, feasibility, and overall assessment. The rating results were described in terms of medians and inter-quartile ranges and were emailed to each panelist prior to the round 2 meeting, along with the results of their own ratings.

Round 2 involved a one-day-long online meeting, where panelists’ comments on the candidate QIs were shared. Panelists responded to questions and comments from other panelists and reflected on their own scores from round 1. During the discussion, panelists were asked to revise the candidate QIs and add new candidate QIs as needed.

After the round 2 meeting (held online), participants were asked to independently re-score the candidate QIs, including the modified and added indicators. As a result of this round 3 scoring, indicators with a median score of 7 or higher and a score of less than 3 by two or fewer panelists were adopted as the final QIs.

### Pilot practice test

A pilot practice test was conducted to assess the feasibility and applicability of the developed QIs to actual clinical measurement. Two facilities with different volumes of surgeries were selected for the pilot test because the content of preoperative and postoperative rehabilitation varies, depending on the surgery volume [12]. In 2023, 271 TKA surgeries were performed at Facility A (expected length of hospital stay: 35 days) and 61 at Facility B (expected stay: 21 days). The eligibility criteria were patients who underwent TKA surgery at either hospital between November 1, 2022 and October 30, 2023 and received rehabilitation before and after surgery. Data were retrospectively collected from the electronic medical records at each facility to determine the extent of implementation of each developed QI at each facility. However, QI 1 “Before surgery, a physical therapist should be aware of patient information, such as age, sex, comorbidities (e.g., obesity, diabetes), and radiographic characteristics,” and QI 13 “A physical therapist should avoid the routine use of continuous passive motion (CPM)” were excluded from the pilot test because they could not be determined from a retrospective electronic medical record survey. For each of the 22 QIs (with the aforementioned two removed), the percentage of patients who underwent rehabilitation as stipulated by the indicator (excluding patients for whom an obvious reason for not implementing the process defined by the indicator was documented) was calculated as the performance of each QI. The facilities were asked to provide feedback on the feasibility of the QIs in actual clinical measurement. Moreover, if a QI was found to have a low performance, i.e., less than 10%, the reason for the poor performance was investigated, considering the possibility of low feasibility. This study was approved by the authors’ affiliated institutions. Informed consent was obtained at each hospital using the opt-out method.

## Results

### Quality indicators

Eight panelists were clinical experts (clinical experience: less than 10 years, *n* = 2; 10–19 years, *n* = 4; and more than 20 years, *n* = 2), and one was a researcher specializing in knee arthroplasty (research experience: 17 years). The mean age of the panelists was 39.7 (range: 28–52) years. The characteristics of the panelists’ institutional affiliations are presented in Table 1. All panelists completed the entire modified Delphi process from rounds 1 to 3.

**Table 1:** Characteristics of the institutional affiliations of the panelists

All panelists (*n* = 9)	
Primary affiliation, *n* (%)	
Regional/district hospital	7 (77.8)
University hospital	1 (11.1)
University	1 (11.1)
Clinician panelists (*n* = 8)	
Clinical work settings, *n* (%)	
Inpatient acute and post-acute phases	1 (12.5)
All phases	7 (87.5)
Volume of TKA patients at each facility, *n* (%)	
< 100 patients/year	2 (25.0)
100–199 patients/year	2 (25.0)
200–299 patients/year	2 (25.0)
> 300 patients/year	2 (25.0)
Expected length of hospital stay after TKA, *n* (%)	
< 10 days	1 (12.5)
10–19 days	2 (25.0)
20–29 days	4 (50.0)
> 30 days	1 (12.5)

TKA: total knee arthroplasty

The 49 QIs rated in round 1 were discussed at the round 2 meeting (held online), resulting in revision of six of the 49 indicators; “A physical therapist or another member of the rehabilitation team should assess and document the quality of life (QOL) before surgery” and “Postoperative assessment of the QOL should be documented by a physical therapist” was merged with “Before surgery and at regular intervals after the acute-care phase, a physical therapist or another member of the rehabilitation team should assess and document patient-reported outcomes, including disease-specific and general QOL measures.” “Before surgery, a physical therapist or another member of the rehabilitation team should provide instructions regarding assistive devices, such as walking aids, and guidance on the home setup and document these interventions” was revised to “A physical therapist or another member of the rehabilitation team should provide instructions regarding assistive devices, such as walking aids, and guidance on the home setup, both preoperatively and postoperatively, as needed, and document these interventions” because it is possible to provide post-surgical instructions and guidance after accounting for the length of hospital stay in Japan. “In the acute-care phase and beyond, a physical therapist should assess and document the balance ability and risk of falling” was modified to “In the acute-care phase and beyond, a physical therapist should assess and document the balance ability and risk of falling, using not only standard assessment batteries but also other appropriate methods as needed.” “Early discharge from the hospital as soon as possible after surgery” was modified to “A physical therapist and other members of the rehabilitation team should facilitate the patients' achievement of the activities of daily living (ADLs) necessary for discharge within the planned length of hospital stay.” Additionally, “A physical therapist or another member of the rehabilitation team should document the patient’s goals for work, sports, and other life activities before surgery” and “A physical therapist or another member of the rehabilitation team should assess and document their achievement of the goals for work, sports, and other life activities postoperatively” were added as new indicators during the consensus process because it is difficult to identify specific goals for work, sports, and other life activities using an assessment battery.

After the online round 2 meeting, the panelists were asked to re-rate the 49 QIs, including six revised indicators, and rate two new additional indicators in round 3. As a result of the round 3 rating, 15 indicators were rejected and 36 indicators were finally adopted. Among these 36 indicators, some had overlapping elements, so they were consolidated and organized into 24 indicators: three for Patient Information; five for Assessment for pain, knee function, and physical functions; two for Preoperative Interventions; nine for Acute-Care Interventions; two for Post-Acute Care Interventions; and three for Others (Table 2).

**Table 2:** Quality indicators for rehabilitation before and after total knee arthroplasty

**Patient Information**	
1	Before surgery, a physical therapist should be aware of patient information, such as age, sex, comorbidities (e.g., obesity, diabetes), and radiographic characteristics
2	A physical therapist or another member of the rehabilitation team should gather and document information regarding family support, social support, and the home setup
3	A physical therapist or another member of the rehabilitation team should document the patient’s goals for work, sports, and other life activities before surgery and assess their achievement postoperatively
**Assessment of pain, knee function, and physical functions**	
4	Across the entire TKA continuum—from preoperative to acute, and beyond—a physical therapist should assess knee pain using a standardized tool, such as the visual analog scale, numerical rating scale, face pain scale, or a scale included in a patient-reported outcome measure, and document these assessments
5	Across the entire TKA continuum—from preoperative to acute, and beyond—a physical therapist should assess knee function using standardized tools, such as knee range of motion (ROM) measurement, knee extension strength assessment, and knee circumference measurement, and document these assessments
6	Across the entire TKA continuum—from preoperative to acute, and beyond—a physical therapist should assess physical function using standardized performance-based tests, such as walking speed measurement, the Timed Up and Go test, and 6-min walk test, and document these assessments
7	In the acute-care phase and beyond, a physical therapist should assess and document the balance ability and risk of falling, using not only standard assessment batteries but also other appropriate methods as needed
8	Before surgery and at regular intervals after the acute-care phase, a physical therapist or another member of the rehabilitation team should assess and document patient-reported outcomes, including disease-specific and general quality of life (QOL) measures
**Preoperative Interventions**	
9	A physical therapist or another member of the rehabilitation team should provide instructions regarding assistive devices, such as walking aids, and guidance on the home setup, both preoperatively and postoperatively, as needed, and document these interventions
10	A physical therapist should provide instructions for exercise and patient education, including an explanation of the postoperative course and lifestyle advice such as pain management, and document these interventions
**Acute-Care Interventions**	
11	A physical therapist or another member of the rehabilitation team should initiate rehabilitation and mobilization within 24 h of surgery and document these interventions
12	A physical therapist should provide and document supervised exercise therapy, including ROM exercises, strength training, and balance and gait training
13	A physical therapist should avoid the routine use of continuous passive motion (CPM)
14	A physical therapist should provide and document neuromuscular electrical stimulation (NMES) to improve muscle strength and function
15	A physical therapist should provide and document cryotherapy for pain management when appropriate
16	A physical therapist should provide and document transcutaneous electrical nerve stimulation (TENS) for pain management whenever appropriate
17	A physical therapist or another member of the rehabilitation team should instruct and document postoperative knee flexion at rest during the first 7 days
18	A physical therapist and other members of the rehabilitation team should facilitate patients' achievement of the activities of daily living (ADLs) necessary for discharge within the planned length of hospital stay
19	A physical therapist should provide general guidance for living at home and a home exercise program after discharge
**Post-Acute Care Interventions**	
20	A physical therapist or another member of the rehabilitation team should regularly verify and document the patient's adherence to the home exercise program for the first 6–8 weeks after surgery
21	A physical therapist or another member of the rehabilitation team should provide and document instructions or interventions to promote increased physical activity
**Others**	
22	A physical therapist or another member of the rehabilitation team should implement clinical pathways throughout the TKA continuum, from the preoperative to postoperative phases
23	A physical therapist or another member of the rehabilitation team should prepare and document a rehabilitation summary for each transfer to another hospital or facility
24	A physical therapist or another member of the rehabilitation team should assess and document patient satisfaction with the rehabilitation program

### Pilot practice test

The pilot practice test was conducted at two hospitals and involved 352 patients. Feedback from both facilities indicated that the QIs were easy to interpret, with no measurement or feasibility issues reported. The results of this test are shown in Table 3 and indicate that the median performance of the QIs was 86.1 (IQR, 56.1–100). However, in Facility A, performance was below 10% for QI 14 (“using neuromuscular electrical stimulation”) and QI 16 (“using transcutaneous electrical nerve stimulation”), both of which demonstrated a performance rate of 0%. This was due to the lack of implementation of these interventions at Facility A, resulting in 0% performance for both indicators. In Facility B, performance rates were below 10% for QI 17, QI 21, QI 22, and QI 24, with QI 21, QI 22, and QI 24 each showing 0% performance. The low performance of QI17 at Facility B was due to failure to instruct the patients to keep the operated knee flexed at rest during the first 7 days. For QI 21, Facility B did not provide instructions or interventions to promote increased physical activity, leading to a 0% performance rate. Additionally, QI 22 yielded a 0% rate as Facility B did not implement a defined clinical pathway. Furthermore, Facility B did not assess patient satisfaction with the rehabilitation process for QI 24, which also scored a 0% performance rate. In Facility A, QI 24 was not applicable, as all participants were discharged home and followed up at Facility A.

**Table 3:** Results of the pilot practice test

Category	QI No	Facility A Performance (%)	Facility B Performance (%)
Patient Information	QI 2	100.0	100.0
	QI 3	86.1	86.0
Assessment of Pain and Function	QI 4	88.5	55.0
	QI 5	92.3	86.5
	QI 6	92.3	80.1
	QI 7	88.4	72.8
	QI 8	89.0	53.2
Preoperative Interventions	QI 9	100.0	100.0
	QI 10	98.5	14.0
Acute-Care Interventions	QI 11	97.3	100.0
	QI 12	100.0	100.0
	QI 14	0.0	45.6
	QI 15	100.0	100.0
	QI 16	0.0	64.9
	QI 17	86.1	1.8
	QI 18	100.0	84.2
	QI 19	100.0	66.7
Post-Acute Care Interventions	QI 20	11.9	71.9
	QI 21	70.1	0.0
Others	QI 22	100.0	0.0
	QI 23	N/A^1^	57.1
	QI 24	61.9	0.0

N/A, not applicable

^1^QI 23 was not applicable to Facility A because all participants were discharged home and received follow-up at Facility A

## Discussion

This study proposed QIs for pre- and post-TKA rehabilitation using the widely-recognized modified Delphi method. The initial 36 QIs were consolidated into 24 items to streamline the indicators and enhance their clarity and usability. The QIs were developed and categorized into Patient Information; Assessment for pain, knee function, and physical function; Preoperative Interventions; Acute-Care interventions; Post-Acute Care interventions; and Others, covering the entire rehabilitation continuum, from preoperative care to post-acute interventions and beyond. The results of the pilot practice test indicated that while most QIs were measurable in real-world clinical practice, four QIs could not be measured by a retrospective review of electronic medical records. The results of the pilot test showed high performance of many QIs, along with low performance, including 0%, of some QIs.

Although the QIs proposed in this study are consistent with previously reported QIs in many respects, including preoperative and postoperative pain assessment, as well as knee and physical function assessments, some differences unique to Japan were identified. For example, preoperative weight management was not included in the QIs developed in this study, although clinical guidelines in the USA [3] and QIs in Canada [18] advocate for preoperative weight management. In the USA, 42.5% of individuals aged 20 years or older are reported to be obese (body mass index [BMI] of 30.0 kg/m^2^ or above) [28], whereas in Japan, only 4.5% of people aged 15 years or older have a BMI of 30.0 kg/m^2^ or above [29]; therefore, weight management was not adopted as a minimum QI for preoperative rehabilitation. Furthermore, the length of hospital stay after TKA in Japan is approximately 21 days [19], which is longer than that in the USA and Canada [20]. Therefore, the authors proposed “Early discharge from the hospital as soon as possible after surgery” as the original QI; however, although the panelists agreed on the need to reduce the duration of hospitalization after TKA in Japan, the difficulty in defining “early” and “as soon as possible” engendered the revision, “A physical therapist and other members of the rehabilitation team should facilitate patients' achievement of the activities of daily living (ADLs) necessary for discharge within the planned length of hospital stay.” Furthermore, the QIs developed in this study did not include tele-rehabilitation or rehabilitation using smartphones or virtual reality, despite increasing interest in these approaches in recent years. Several systematic reviews have demonstrated that tele-rehabilitation can effectively reduce pain and improve physical function after TKA [30, 31]. However, other studies have highlighted that the evidence supporting these methods remains of low to moderate quality or is otherwise limited [32–34], with ongoing challenges yet to be addressed. Consequently, tele-rehabilitation was not deemed suitable for implementation as a standard approach to rehabilitation.

Although a previous study reported the development of QIs for pre- and post-TKA rehabilitation [18], no studies examined the real-world clinical measurement using QIs for pre- and post-TKA rehabilitation. This study conducted a pilot test and found that some QIs performed poorly (including 0%). However, the feedback indicated that this poor performance could be attributed to the low proportion of patients receiving rehabilitation, as stipulated by the indicators in this pilot test, and not to the low feasibility or applicability of the QIs developed in this study. Further studies are required to verify the feasibility and applicability of clinical measurement using the QIs developed in this study.

It has been suggested that there exists an evidence–practice gap in rehabilitation after TKA in Japan [12]. The QIs developed in this study can be used to measure the evidence–practice gap and guide quality improvement initiatives. The QIs in this study enable physical therapists and rehabilitation team members to systematically acquire essential patient information and assess pain, knee function, and physical function. In addition, the QIs define specific intervention content for the acute-care phase, thus facilitating the delivery of standardized rehabilitation to patients. Adherence to these QIs would ensure that the rehabilitation staff provide a comprehensive, evidence-based rehabilitation program that is consistent with the established standards in real-world clinical practice.

This study has several strengths. Panelists are an important element in the formulation of high-quality QIs. As previous studies have reported that the content of pre- and post-TKA rehabilitation in Japan varies depending on the volume of surgeries [12], the panel members included in this study were not only selected from different regions of Japan, but also involved experts who belonged to institutions with varying volumes of surgeries. Moreover, the QIs developed in this study demonstrated the importance of continuous rehabilitation from the preoperative phase through the acute and post-acute phases. In Japan, hospitals are being functionally differentiated into advanced acute care, acute care, convalescent care, and chronic care centers [35], and the importance of continuous rehabilitation demonstrated in this study suggests the need for each facility to fulfill its designated role and cooperate with others according to their respective functions.

However, this study is also subject to some limitations. First, it was not possible to include patients among the panelists. Future QIs would benefit patients more if their opinions were included during QI development. In addition, the QIs developed in this study were limited to TKA for knee osteoarthritis. Therefore, their utility for TKA for rheumatoid arthritis and osteonecrosis of the femur is unknown and needs to be verified in the future. Finally, the pilot practice test was conducted at only two facilities. Future studies should conduct broader practice tests to ensure the generalizability of the QIs developed in this study.

## Conclusion

This study developed QIs related to pre- and post-TKA rehabilitation and confirmed the measurability of these QIs in a pilot practice test. Future research should use these QIs to assess and address gaps in rehabilitation care.

## Data Availability

The datasets used and/or analyzed during the current study are available from the corresponding author on reasonable request.

## Abbreviations

TKA: Total knee arthroplasty
CPM: Continuous passive motion
QI: Quality indicator
SR: Systematic review
QOL: Quality of life
ADLs: Activities of daily living
